# Therapeutic advances in anaplastic thyroid cancer: a current perspective

**DOI:** 10.1186/s12943-018-0903-0

**Published:** 2018-10-23

**Authors:** Shikha Saini, Kiara Tulla, Ajay V. Maker, Kenneth D. Burman, Bellur S. Prabhakar

**Affiliations:** 1 Department of Microbiology and Immunology, University of Illinois-College of Medicine, Chicago, IL USA; 2 Department of Surgery, Division of Surgical Oncology, University of Illinois-College of Medicine, Chicago, IL USA; 3 MedStar Washington Hospital Medical Center, Washington, DC USA; 4 Jesse Brown VA Medical Center, Chicago, IL USA

**Keywords:** Anaplastic thyroid Cancer, Therapeutics, Inhibitors, Tumor, Immunotherapy, Clinical trial

## Abstract

Thyroid cancer incidence is increasing at an alarming rate, almost tripling every decade. In 2017, it was the fifth most common cancer in women. Although the majority of thyroid tumors are curable, about 2–3% of thyroid cancers are refractory to standard treatments. These undifferentiated, highly aggressive and mostly chemo-resistant tumors are phenotypically-termed anaplastic thyroid cancer (ATC). ATCs are resistant to standard therapies and are extremely difficult to manage. In this review, we provide the information related to current and recently emerged first-line systemic therapy (Dabrafenib and Trametinib) along with promising therapeutics which are in clinical trials and may be incorporated into clinical practice in the future. Different categories of promising therapeutics such as Aurora kinase inhibitors, multi-kinase inhibitors, epigenetic modulators, gene therapy using oncolytic viruses, apoptosis-inducing agents, and immunotherapy are reviewed. Combination treatment options that showed synergistic and antagonistic effects are also discussed. We highlight ongoing clinical trials in ATC and discuss how personalized medicine is crucial to design the second line of treatment. Besides using conventional combination therapy, embracing a personalized approach based on advanced genomics and proteomics assessment will be crucial to developing a tailored treatment plan to improve the chances of clinical success.

## Background

Thyroid cancer is the most common endocrine-related malignancy, accounting for more than 90% of endocrine cancers [1]**.** In 2017, more than 56,870 new cases were diagnosed in the United States constituting 3.4% of all new cancer cases [2]. The majority of thyroid tumors are pathologically differentiated cancers and exhibit good prognosis with > 98% five-year survival. Among these differentiated thyroid cancers (DTC), papillary thyroid cancer (PTC) is the most common, comprising about 80% of all thyroid cancers. The other DTCs include follicular thyroid cancer (FTC), and medullary thyroid cancers (MTC). These malignancies originate from follicular and parafollicular cells, respectively. A small subset of thyroid cancer, known as anaplastic thyroid cancer (ATC), is undifferentiated, and nearly incurable with a median survival of only six months. Because of its dismal prognosis, it is responsible for 40–50% of total thyroid cancer-related deaths in the United States. Poorly differentiated thyroid cancer (PDTC) resembles ATC due to its aggressive nature but has partial overlap with FTC/PTC, retaining follicular elements and thyroglobulin production [3]. Thus, as opposed to most DTCs for which surgery and radioiodine therapy will result in an excellent prognosis, ATC poses a significant clinical challenge as it is highly aggressive and with comparably no effective therapeutic options.

According to the American Thyroid Association (ATA) guidelines, first-line treatment for ATC includes surgical resection, if possible, and external beam radiation therapy for local control. Though total thyroidectomy with high-dose radiation therapy is associated with improved survival [4]; second-line treatment with targeted therapies, single or in combination, are often employed. Thus, current clinical ATC management is still evolving, and new treatments are being urgently developed. This review provides comprehensive information related to therapeutic options that are available or in the pipeline. A comprehensive review of original research articles, reviews, clinical investigations, and editorials published in the last ten years from Medline/PubMed, Google Scholar and SciFinder was conducted. Clinical trials related information was assessed from https://clinicaltrials.gov/. The purpose of this study is to highlight current treatment strategies and their limitations, improve our understanding of their pitfalls, and to propose ways to overcome these hurdles.

## Clinical manifestation and diagnosis

ATC often presents as a neck mass causing dysphagia, dysphonia or hoarseness, stridor, and dyspnea due to mass effect on the esophagus and the trachea [5, 6]. According to the Union for International Cancer Control (UICC) staging system, ATC tumors are automatically designated as stage IV disease irrespective of tumor burden and presence or absence of metastasis. They are sub-classified as IVa, IVb, IVc and IVd depending on the extent of invasion of the surrounding tissue layers [7]. Pathologically, ATC cells are spindle-shaped, giant and squamoid cells, with a high mitotic index, necrosis, hemorrhage and vascular invasion [8]. Due to their vascularity, misdiagnosis with angiosarcoma is common. About 70% of ATCs invade surrounding tissues including fat, trachea, esophagus and larynx. The most common metastatic sites in ATC patients are the lungs, bone, and brain [8].

The diagnosis of ATC is confirmed via fine needle aspiration (FNA). However, false-negatives are common due to low cellularity and inflammatory and necrotic debris. At the same time, there is a lack of definitive molecular biomarkers to detect ATC in FNA biopsies with high sensitivity. To improve the sensitivity and specificity of diagnosis, molecular diagnostics using customized panels based on next-generation sequencing holds promise for the future [9, 10].

## Current therapeutic regimes

**Fig. 1:**
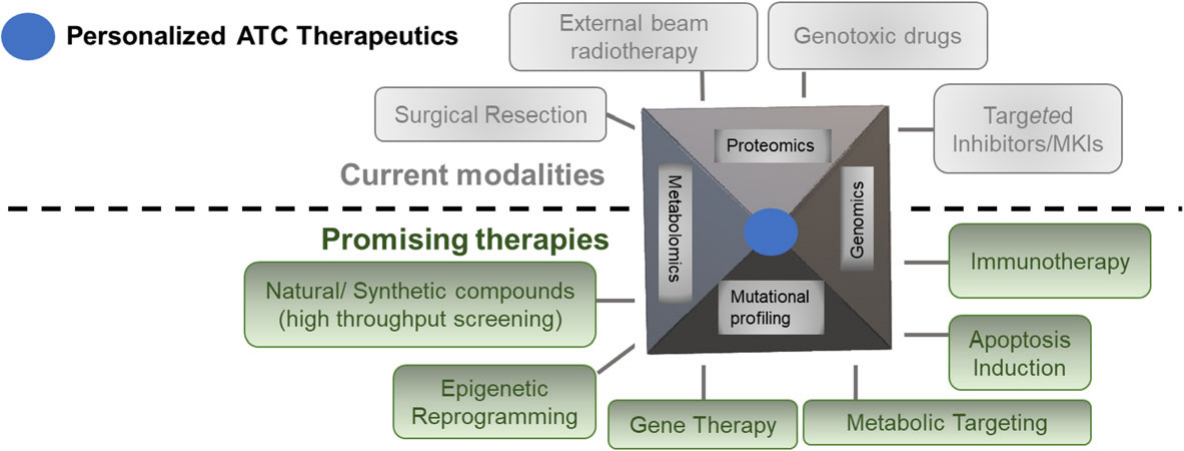
Current and promising therapeutics that can be employed for personalized medicine development: Clinically, ATC therapeutic regime involves use of more than one modality shown above (current therapies, depicted in grey color). Several promising categories of drugs were explored for their therapeutic implications and can be employed in single or combination with other drugs (depicted in green color). However, the best strategy would be to evaluate and design the personalized treatment plan by determining underlying mutations, genetic lesions, oncogenic signaling cascades and other metabolomic “Achilles heels”

For ATC, a multimodal therapeutic approach is employed which includes surgical resection, hyper-fractionated accelerated external beam radiotherapy, followed by a combination of chemotherapies and/or palliative care [11]. Despite multi-modality treatment, the prognosis for ATC patients is poor. Due to the failure of single-agent chemotherapy, a combination of two or more drugs such as Paclitaxel, Cisplatin, Doxorubicin, Pegfilgrastim and Docetaxel is administered to ATC patients [12]. Second-line treatments include more targeted therapies such as tyrosine kinase inhibitors, anti-angiogenic drugs, and agonists and multi-kinase inhibitors targeting hyperactive BRAF, mTOR, and/or BCR-ABL. In the past decade, several novel drugs that target proliferation, angiogenesis, immunosuppression, metabolomic changes and epigenetic reprogramming have been evaluated (Fig. 1). However, it is pertinent to note that clinical trials of potentially effective treatments for ATC are hampered by its low incidence and aggressiveness that limits enrollment leading to poor statistical power and a limited treatment time-frame. Current mainstays of ATC therapeutic management include:

### Surgical resection

Surgical management is generally precluded for patients with ATC due to extensive metastasis [13]; however, complete surgical resection when achievable with limited morbidity is recommended for localized disease. If the tumor is limited to the thyroid parenchyma, thyroidectomy with wide margins is recommended, especially if the tumor reflects good prognostic features including unilobar disease, diameter < 5 cm, and without nodal spread, for which lobectomy alone may be performed [14]. In fact, in the rare instance of a small, unilocular, contained mass, total thyroidectomy appears to offer no survival advantage over lobectomy while carrying greater operative risks [15, 16]. Interestingly, in a Mayo Clinic series over 50-years, incomplete resection was no worse than negative margins, in regard to overall patient survival. This is likely due to the overall poor prognosis and difficulty in local control even with surgery for this aggressive disease. Regardless, many still advocate for total thyroidectomy and central node dissection, and current guidelines recommend this approach if R_0_ (microscopically negative resection) or R_1_ (grossly negative, microscopic positive) resection can be achieved [12, 16].

Complete resection is a challenge, often due to tumor size, extra organ growth extension, and local invasion. The goal of surgery is for a margin-negative R_0_ resection. Pre-operative evaluation with high-quality fine-cut cross-sectional imaging and ultrasound is necessary to determine tumor extent and the possible local involvement of the carotid artery, jugular vein, vagus nerve and its branches, trachea, esophagus, sternocleidomastoid and strap muscles. Speech changes may already be evident at the time of presentation, which is concerning for recurrent laryngeal nerve involvement. Laryngoscopy can be an invaluable tool to assess vocal cord mobility while bronchoscopy allows visual assessment for tracheal involvement. If one recurrent laryngeal nerve is already involved, then it is of utmost importance that the contralateral nerve is preserved and/or only ipsilateral lobectomy is performed, if possible. If a contralateral resection is necessary to obtain negative margins, the use of a nerve stimulator may improve recurrent laryngeal nerve identification during surgery, although this is surgeon-dependent. Concurrently, imaging is critical in the assessment of metastatic disease, in which case resection should be reserved purely for cases of airway compromise.

After a proper staging assessment, if resection can be performed, patients have improved outcomes even with margin positivity. However, laryngectomy is usually discouraged due to the likelihood of persistent disease and the severe morbidity associated with this procedure. Current recommendations, based on moderate quality evidence, advise for lobectomy, total, or near-total thyroidectomy, with therapeutic lymph node dissection [12]. This approach involves the standard lifting of sub-platysmal flaps and division of the deep cervical fascia in the midline to expose the underlying gland. Involvement of the strap muscles necessitates the division of the muscles, which should be performed en bloc with the thyroid. Due to the desire to obtain wide negative margins, and because of the infiltrative nature of this cancer, the parathyroid glands may need to be sacrificed. In these instances, efforts should be made to identify and preserve all uninvolved parathyroid glands, and to confirm histologically, mince, and re-implant the tissue to prevent severe hypocalcemia. Central and lateral lymphadenectomy is performed at the same time of surgical resection, and additional soft tissue, fascia, muscle, or veins should be taken en bloc if invaded.

Tracheostomy or palliative resection is sometimes necessary due to tracheal involvement in order to manage airway compromise or esophageal obstruction. As has been mentioned, chemotherapy alone is ineffective in controlling this disease. However, a study by the Swedish Anaplastic Thyroid Cancer Group combined neoadjuvant Adriamycin and 3 weeks of radiation followed by debulking surgery and adjuvant radiation [17]. With this protocol, some previously unresectable tumors became resectable. Though there were individual patient successes, overall improvement in local control and overall survival remains to be demonstrated. Clearly, surgery has a role in the treatment of this disease, particularly for small, localized tumors, and in the palliative setting to avoid suffocation; however, optimal outcomes involve multimodality treatment strategies.

### External beam radiation therapy

Due to their undifferentiated phenotype and lack of sodium-iodine symporters, radioactive iodine (I^131^) ablation therapy is ineffective in localizing radiation to the gland and therefore, external beam radiation therapy is utilized. Due to the aggressive time-course of the disease, accelerated, hypo-fractionated radiation treatment is preferred. However, it can induce acute toxicity. The dosing regimens utilized are based on case studies, non-randomized trials, and institutional experience. The contribution of high-intensity external beam radiation therapy to improve survival relies on the ability to adequately resect the tumor, which as mentioned above, is only possible in a minority of cases. A recent meta-analysis including 17 retrospective studies of 1147 patients, showed that postoperative radiotherapy significantly improved survival in patients with resected tumors versus patients with surgery alone (HR, 0.556; 95% confidence interval, 0.419–0.737; *p* < 0.001). Moreover, it was also found that patients with stage IVa (HR, 0.364; *p* = 0.012) and IVb (HR, 0.460; *p* = 0.059) disease may derive a survival benefit from post-operative radiotherapy, whereas stage IVc patients may not [18]. A meta-analysis of 1288 ATC patients from National Cancer Database revealed that patients with unresected ATC might benefit from multimodal loco-regional treatment that incorporates higher (60–75 Gy) versus lower (45–59.9 Gy) radiation doses [19]. The major challenge in analyzing the advantages of adjuvant radiation is the inherent time bias, as many patients studied already had extremely limited survival at the time of diagnosis. Recently, a unique single platform dual therapy based on polyethylene glycol-coated [64Cu] CuS nanoparticles was developed, which combines radiation and photothermal therapy. This investigation showed promising improvement in overall survival in a preclinical orthotopic mouse model and has the potential for clinical translation [20].

Attempts have also been made to re-sensitize ATC cells to radioiodine therapy by reintroducing sodium iodide symporters. In this context, a recent study showed promising results by employing nanoparticle vectors (polyplexes) based on linear polyethylenimine (LPEI) and polyethylene glycol (PEG). These nanoparticles were coupled to the synthetic peptide GE11, which acts as an EGFR ligand and provides the basis for directed delivery. These nanoparticles effectively induced sodium-iodide symporters in ATC cells with high EGFR expression [21].

### Chemotherapy

As per National Comprehensive Cancer Network guidelines, for stage IVa/ IVb ATC tumors total thyroidectomy (if possible) should be followed by external beam radiotherapy and systemic therapy with genotoxic drugs such as Paclitaxel, Doxorubicin, Docetaxel, Carboplatin, Dabrafenib and Trametinib. Recommended regimens include Paclitaxel and Carboplatin combinations, Docetaxel and Doxorubicin combinations, Paclitaxel alone, or Doxorubicin alone [12]. For BRAFV600E mutation-positive tumors, a combination of Dabrafenib and Trametinib has shown promising clinical responses and is recommended [22]. For stage IVc patients, in addition to these regimens, palliative radiation therapy for locoregional metastases and second-line treatment with various systemic chemotherapies are often considered. Though Paclitaxel is the most effective chemotherapeutic drug, chemo-resistance is common, mainly due to tumor-associated macrophages (TAMs) in ATC. TAMs occupy 50% of the tumor volume and provides paracrine signals via CSF-1/CSF-1R axis, which promotes tumor progression [23]. Thus, targeting CSF-1/CSF-1R pathway in TAMs was shown to restore the sensitivity of thyroid cancer cells to Paclitaxel [24]. Along similar lines, JAK/STAT inhibitors can also be employed for Paclitaxel-resistant tumors [25]. Such therapeutics based on the mutational and proteomic landscape of tumors might be helpful in overcoming therapeutic resistance in ATC.

### Targeted/multi-targeted inhibitors

Targeted inhibitors act on a specific target molecule and prevent cancer growth and progression. These inhibitors usually target hyperactive or mutant molecules active in signaling pathways in cancer cells. RAF/MAPK signaling is integral to ATC progression and some of the molecular therapeutics including the BRAF inhibitor (Vemurafenib/PLX4032) and the MEK inhibitor (Selumetinib/AZD6244) target this signaling cascade. In general, these single-targeted inhibitors failed to show significant therapeutic responses in ATC patients leading to the use of multi-kinase inhibitors (MKIs). MKIs can simultaneously act on two or more targets. MKIs such as Sorafenib, Axitinib, Pazopanib and Sunitinib has been evaluated in preclinical models, as well as in clinical trials, and have shown some encouraging results (Table 1). Sorafenib demonstrated progression-free survival (PFS) of about five months in phase III clinical trial with patients exhibiting manageable toxicities when compared to the placebo group [26]. However, most of the patients experienced disease relapse indicating the need for a combination of MKIs to improve outcomes. In this context, in advanced DTC patients who were unsuccessfully treated with Sorafenib, salvage therapy using Sunitinib, Pazopanib, Cabozantinib, Lenvatinib, and Vemurafenib was tested. With salvage therapy, a partial response (PR) was observed in 41% (7/17) patients suggesting a synergy of these drugs with Sorafenib [27]. In an attempt to improve the specificity of Sorafenib, it was chemically loaded in Poly-lactic-co-glycolic acid (PLGA) nanoparticles and combined with Cetuximab (EGFR inhibitor) [28]. This formulation significantly improved cytotoxicity against ATC cells without affecting normal thyroid cells.

**Table 1:** Results from clinical trials conducted in advanced, metastatic, radioiodine-refractory and anaplastic thyroid cancers conducted between 2013 and 2017 in the United States (Source: https://clinicaltrials.gov/)

No.	Drug	Phase	Cancer	Number of patients	Response Rate	Progression free survival	Overall survival	Reference
1	Sorafenib (Bay43–9006, Nexavar)	II	ATC	20	PR in 2/20 (10%); Stable disease in 5/20 (25%)	1.9 months	–	[107]
2	Carbozantinib	III	MTC	330	28%	11.2	–	[108]
3	Efatutazone+ Paclitaxel	I	ATC	15	PR = 1; SD = 7	3.3 months	–	[39]
4	Pazopanib	II	Advanced and progressive medullary	35	5/35	9.4	19.9	[109]
5	Fosbretabulin + Paclitaxel/Carboplatin	II	ATC	8	20%	3.3	5.2 months	[110]
6	Vemurafenib		BRAFV600E positive, metastatic, radio-iodine refractory PTC	26	10/26	–	–	[110]
7	Axitinib	II	Advanced thyroid cancer	52	35%	16.1	23.2	[111]
8	Levatinib	III	Iodine refractor TC	261	64.8%	18.3	–	[112]
9	Sunitinib (second line of therapy)	II	Progressive, radio-iodine Refractory thyroid cancer	25	5/20 (25%)	6 months	13 months	[113]
10	Cabozantinib (XL-184)	III	Advanced MTC			11.2 months	–	[114]
11	Dabrafenib plus trametinib	II	BRAF V600E–mutated anaplastic thyroid cancer	16	69%	–	–	[23]

Another MKI, Lenvatinib, was evaluated in a phase III trial in metastatic DTCs with a 65% response rate (RR), and a median PFS of 18.3 months versus 3.6 months in placebo-treated patients [29]. A retrospective analysis of ATC patients treated with Lenvatinib resulted in partial responses (PR) in 60% (3/5) of patients whereas 40% (2/5) experienced stable disease with manageable toxicity. In addition, the observed objective response rate (ORR) was 60% with median time to progression (TTP) and overall survival (OS) as 88 and 165 days respectively [30].

A very recent clinical trial in BRAFV600E–mutated ATCs showed positive results by using a combination of Dabrafenib (150 mg twice daily) and Trametinib (2 mg once daily). Prior to the treatment, all patients received either surgery or radiation therapy. The overall response rate (RR) was 69% (11 of 16; 95% CI, 41% to 89%). Predicted 12-month estimates of median duration of response, PFS and OS were 90%, 79%, and 80%, respectively. So far, this is the first clinical trial that showed a high clinical response for this orphan disease [22].

### Ongoing clinical trials

Some potential MKIs have been tested in clinical trials and a list of recently completed clinical trials in advanced thyroid cancers is outlined in Table 1. In advanced FTC, encouraging results were obtained by using the selective allosteric MEK1 and MEK2 inhibitor Selumetinib (AZD6244, ARRY-142886). Particularly, Selumetinib resensitized radio-iodine refractory ATC to uptake radio-iodine and can be used as a complementary therapy [31]. Targeting mTOR signaling holds promise and a phase I/II clinical trial evaluating the efficacy of mTOR inhibitor, Sapanisertib is underway. Several clinical trials are also underway to assess the combination of Selumetinib, Everolimus, Lenvatinib, Cabozantinib, Vandetanib and Vatalanib in ATC patients [32]. A recently completed phase II/III clinical trial using a combination of Paclitaxel and Valproic acid (VPA) showed no benefit in overall survival and disease progression [33]. Four drugs that have been approved for advanced thyroid cancer treatment after phase III clinical trial completion include Vandetanib (ZETA), Cabozantinib (EXAM), Sorafenib (DECISION) and Lenvatinib (SELECT) [34, 35]. Of note, several MKIs such as Sorafenib, Axitinib, and Sunitinib exhibited limited efficacy [36, 37]. Aimed at improving the Paclitaxel efficacy, a phase I clinical trial using a combination of Paclitaxel and the PPARγ agonist, Efatutazone demonstrated safety and merited further evaluation in phase II in ATC patients which is ongoing (Table 2) [38].

**Table 2:** Ongoing Clinical Trials in Anaplastic Thyroid Cancer (as on July 15, 2018), listed from https://clinicaltrials.gov/

S. No	Phase	Drug	Drug Action	Clinical Trial No.	Status	Sponsors
1	II	MLN0128	mTOR kinase inhibitor	NCT02244463	Recruiting	Dana-Farber Cancer Institute, USA
2	II	Lenvatinib	MKI against VEGFR1, 2, and 3	NCT02726503	Recruiting	Translational Research Informatics Center, Kobe, Hyogo, Japan
				NCT02657369	Recruiting	Eisai Inc. USA
3	Early phase I	Trametinib in combination with Paclitaxel	MEK inhibitor (Trametinib) with chemotherapy	NCT03085056	Recruiting	Memorial Sloan Kettering Cancer Center, USA
4	II	Pembrolizumab	Antibody against PD-1 receptor	NCT02688608	Recruiting	University of Texas Southwestern Medical Center, USA
5	II	Inolitazone Dihydrochloride (Efutazone) and Paclitaxel	PPAR-γ agonist (Efutazone) with chemotherapy	NCT02152137	Recruiting	Alliance for Clinical Trials in Oncology, USA
6	I	Combination of Durvalumab (MEDI4736) or Tremelimumab with Stereotactic Body Radiotherapy (SBRT)	Checkpoint inhibitor drugs: Durvalumab (PD-1/PDL-1 interaction blocker) and Tramelimumab (anti-CTLA4 antibody) with radiations	NCT03122496	Recruiting	Memorial Sloan Kettering Cancer Center, USA
7	II	Intensity-Modulated Radiation Therapy and Paclitaxel with or Without Pazopanib Hydrochloride	Pazopanib is a MKI against c-kit, FGFR, PDGFR and VEGFR	NCT01236547	Ongoing but not yet recruiting participants	National Cancer Institute (NCI), USA
8.	II	Ceritinib	ALK inhibitor	NCT02289144	Recruiting	University of Texas Southwestern Medical Center, USA
9	II	Atezolizumab Combinations with or without chemotherapy such as paclitaxel, Vemurifinib, Nab-paclitaxel, Cobimetinib and Bevacizumab	anti-PDL-1 antibody (Atezolizumab)	NCT03181100	Recruiting	M.D. Anderson Cancer Center, USA
10	I	FAZ053 as Single Agent and in combination with PDR001	FAZ053 is anti-PDL-1 antibody and PDR001 is monoclonal antibody against PD-1.	NCT02936102	Recruiting	Novartis Pharmaceuticals, USA
11	II	Dabrafenib and Trametinib	Dabrafenib acts against BRAFV600E mutations and Trametinib is MEK (1 and 2) inhibitor	NCT02034110	Recruiting	GlaxoSmithKline, USA
12	II	GW 786034 (Pazopanib Hydrochloride)	Pazopanib is a MKI against c-kit, FGFR, PDGFR and VEGFR	NCT00625846	Active, not recruiting	National Cancer Institute (NCI), USA
13	II	Pembrolizumab, Chemotherapy,and Radiation Therapy With or Without Surgery	anti-PD1 immunotherapy	NCT03211117	Active, not recruiting	Mayo Clinic,National Cancer Institute (NCI), USA
14	I/II	PDR001	anti-PD1 monoclonal antibody	NCT02404441	Recruiting	Novartis Pharmaceuticals

## Promising therapeutic options

Therapeutic success in ATC patients has been very limited and thus, there is a continuing need to develop novel therapies. Several pre-clinical investigations have been carried out to explore the potential of various drugs, and some of the promising categories are discussed below:

### Aurora kinase inhibitors

Aurora kinases are serine/threonine kinases involved in chromosomal segregation and cytokinesis during mitosis. These kinases include three members: Aurora A, B and C. Besides mitosis, these are involved in determining cell polarity, migration and invasion, and telomerase activity [39]. Their dysregulation is frequently noted in ATC as compared to PTC or normal thyroid tissues. [40]. Several Aurora kinase inhibitors including MK-0457 (VX-680), SNS-314 Mesylate, ZM447439, and AZD1152 have been tested and have shown significant cell cycle arrest and subsequent reduction in growth and proliferation in vitro. Particularly, administration of MLN8054 reduced tumor volume by 86% in an ATC xenograft mouse model [41]. In a different study, a combination of MLN8054 with Bortezomib (proteasome inhibitor) induced apoptosis and cell cycle arrest in ATC cells [42]. Another Aurora kinase inhibitor, Pazopanib showed synergistic cytotoxicity with Paclitaxel [43].

Another member of mitosis-related kinases, Polo-like kinase-1 (PLK-1), which regulates chromosomal segregation, is highly active in ATC. Its inhibitor, GSK461364 induced apoptosis in both ATC allograft mouse model and PDTC-derived cell lines [44].

### Natural/synthetic compounds

Screening of potential drugs using compound libraries has resulted in the identification of several novel inhibitors. In a high throughput screening of 3282 drugs targeting mTOR, Torin2 showed a remarkable reduction in cellular proliferation in vitro and inhibition of tumor growth and metastasis in vivo [45]. In another study, pretreatment with Carfilzomib (Proteasome inhibitor) resulted in the reduced metastatic spread and disease progression in mice [46]. Similarly, administration of YM155 (Survivin inhibitor) and CUDC-101 (Histone Deacetylase and EGFR inhibitor) showed a significantly suppressed tumor growth and reduced metastasis in vivo [47, 48]. CUDC-101 is currently underway for testing in phase II clinical trial in ATC patients (Table 2).

As these tumors do not respond to radio-iodine ablation therapy, several compounds have been identified by high-throughput screening that can induce re-differentiation programme. An example of such compounds, Resveratrol can induce functional Notch1 protein expression and activate transcription of thyroid-specific genes including *TTF1, TTF2, Pax8*, and *NIS* [49]. In addition, it can also reduce stem cell markers confirming its potential to induce differentiation [50]. Other examples of such re-differentiation-inducing compounds are 1, 25 dihydroxy vitamin D3 (Calcitriol), Hesperetin and VPA [50–52]. Similarly, Chrysin can upregulate the expression of NIS by activating Notch and its downstream effector, HES1. Also, treatment with Chrysin resulted in diminished cellular growth in vitro and tumor growth in vivo [53]. Collectively, these compounds may complement radioiodine therapy and warrant more comprehensive assessment to explore their clinical efficacy.

### Gene therapy using oncolytic viruses

This therapy is a promising approach to restore the expression of tumor suppressor genes and to target oncogenes. Restoration of NIS and p53 expression using an adenovirus-5 vector showed a significant increase in uptake of radioactive iodine (I^131^) and improved cytotoxicity in vitro and in vivo [54]. The combination of oncolytic viruses with small molecule inhibitors yielded promising preclinical results. For instance, a combination of the ATM (a Ser/Thr kinase involved in DNA replication) inhibitor, KU55933 with oncolytic adenovirus, dl922–947 improved the efficacy of ionizing radiation treatment in ATC cells [55]. Recently, a novel approach using an oncolytic virus (*dl*922–947) showed a remarkable increase in the efficacy of PARP inhibitor, Olaparib [56]. Additionally, the oncolytic adenovirus, dl922–947 was shown to modulate tumor microenvironment by decreasing IL-8/CXCL8 and MCP-1/CCL2 expression which resulted in compromised angiogenesis and macrophage infiltration [57]. Although preclinical studies demonstrated encouraging results, the clinical implications of oncolytic viruses is still evolving [58].

### Novel targeted inhibitors

Specific molecules that can inhibit the key signaling cascades in ATC are of significant interest. In this regard, some of the targeted tyrosine kinases, which are targeted in ATC include EGFR, PDGFR, VEGFR, cMET (Hepatocyte Growth Factor Receptor) and RET [59, 60]. All these molecules have been targeted and resulted in a semi-favorable response in ATC patients. A list of target-specific inhibitors is given in Table 3. Several PI3k/Akt/mTOR pathway inhibitors such as Everolimus, Temsirolimus, GSK69093, MK-2206, PX866, and ZSTK474 have been tested in preclinical and clinical studies. Everolimus showed safety in phase I clinical trial and is currently in phase II clinical trial (NCT02143726) in combination with Sorafenib [61] for the treatment of ATC.

**Table 3:** Different categories of drugs used in preclinical and clinical studies in ATC

Chemotherapeutic agents	
Topoisomerase inhibitor	Doxorubicin, Etoposide
Microtubule assembly	Paclitaxel, Vinorelbine, Docetaxel
DNA crosslinking agents	Cisplatin, Carboplatin, Cyclophosphamide, Neoplatin
Nucleoside Analog	Gemcitabine, 5- fluorouracil
Targeted inhibitors/antibodies	
ALK1	GSK461364A
Akt	MK-2206 2HCL, Perifosine, GSK690693, GDC-0068, AT7867
Aurora Kinases	MK-0457 (VX-680), SNS-314 mesylate, ZM447439, AZD1152 and MLN8054
Bcl2	Obatoclax
CDK	BP14
EGFR	Cetuximab (C225), Manumycin A, Geldanamycin, Gefitinib (ZD1839)
HSP90	Tanespimycin (17-N-allylamino-17-demethoxygeldanamycin, NVP-A0Y922, SNX5422
I-κB	Ciglitazone (upregulates TrailR1, −R2)
PARP	Olaparib
PD-1 receptor	Pembrolizumab, PDR001
PDL-1	Durvalumab, Atezolizumab, FAZ053
CTLA4	Tramelimumab
TGF-β	LY2157299, SB 525334, LY2109761, Perfenidone, GW788388
SMO (Wnt signaling pathway)	LDE225, LY2940680, PF-5274857, SANT-1
γ-secretase	RO4929097, LY-411575
Anti-angiogenic agents	
Vascular disrupting agent	Combretastatin A4 phosphate (CA4P), Fosbretabulin
VEGF	Bevacizumab, AZD2171, Cediranib
Multi-Kinase inhibitors	
VEGF 1, 2 and 3, PDGFR and c-KIT	Axitinib (AG-013736), Pazopanib
VEGFR1, 2 and 3, EGFR and RET kinases	Vandetanib
VEGFR-1, PDGFR, RET, FLT-3 and CSF-1R	Sunitinib
VEGFR2, EGFR and RET	CLM94
BCR-ABL, PDGFR and c-kit	Imatinib
VEGFR 1, 2, PDGFRβ, RET, BRAF and c-Kit	Sorafenib (Bay43–9006, Nexavar)
VEGFR-1, −2 and − 3, PDGFRβ, RET, FGFR −1, − 2, −3, −4 and c-KIT	Lenvatinib (E7080)
VEGFR 2, RET, MET, kit	Cabozantinib
VEGFR −1, − 2, −3, RET, kit, PDGFR	Motesanib
VEGFR − 1, −3, PDGFR, FGFR1–3	Ninetedanib
RET, PDGFR, FGFR, FLT3, kit	Ponatinib
MET, ALK, ROS1	Crizotinib
Epigenetic modifiers	
HDAC inhibitors	Valproic acid, Thailandepsin A (TDP-A), Trichostatin A (TSA), Suberoyl Amide Hydroxamic Acid (SAHA), N-hydroxy-7-(2-naphthylthio)heptanomide (HNHA)
BET inhibitors	JQ1, I-BET762
Miscellaneous	
HDACs, EGFR (dual inhibitor)	CUDC-101
Proteosome inhibitors	Carfilzomib, Bortezomib (PS-341)
PPARγ agonists	Rosiglitazone, RS5444, Pioglitazone, Troglitazone

BRAFV600E inhibitor, Vemurafenib, also showed limited efficacy [62], due to activation of downstream PI3k/Akt and MAPK pathways by alternate mechanisms. For example, cMET can directly activate PDK-1 and Ras thereby, bypassing BRAF mediated signaling and avoiding the current PI3k/Akt/mTOR pathway targets. In an effort to overcome this impediment, downstream pathway inhibitors were explored. An example of such inhibitors is OSU-53 which targets mTOR and activates AMPK. This molecule effectively inhibited cellular growth in ATC cells lines with activating mutations in *Ras* or *BRAF* [63]. Among the newly discovered MKIs, a class of “pyrazolo[3,4-*d*]pyrimidine” compounds (CLM29 and CLM24) that inhibit several targets such as EGFR and VEGFR, showed anti-proliferative and anti-metastatic effects in ATC-derived primary cells and established cell lines [64].

NF-κB signaling is crucial in ATC progression and can be targeted by employing proteasome inhibitors. Carfilzomib, a potent proteasome inhibitor, induced apoptosis in ATC cells by upregulating p27 and downregulating the anti-apoptotic molecule ATF4 [46]. Likewise, Bortezomib in combination with MLN8054 (Aurora kinase inhibitor) showed reduced cellular growth and induced apoptosis in ATC cells [42] Another promising HIV protease inhibitor, Nelfinavir, which blocks both MAPK and PI3k/Akt signaling pathways, exhibited induction of DNA damage and inhibition of cell proliferation in ATC cells in vitro [65].

Heat Shock Proteins (HSPs) represent another class of promising molecular targets for therapeutic purposes. These proteins are expressed in stressful conditions in normal cells but are aberrantly expressed in cancer cells. Combined inhibition of HSP90 (by Radicicol) and HSP70 resulted in significant induction of apoptosis in ATC cells [66]. Two newly discovered HSP90 inhibitors, KU711 and WGA-TA, showed a remarkable reduction in stemness (i.e. aldehyde dehydrogenase (ALDH)^+^ and CD44^+^), migration and invasion of ATC cells in vitro, and downregulation of β-Catenin, BRAF, Akt, and phosphoAkt [67]. These promising findings provide the framework for another therapeutic option to attack this aggressive and debilitating disease.

### Epigenetic silencing

In an intricate process of carcinogenesis, epigenetic reprogramming can lead to activation of tumor-promoting genes and de-activation of tumor-suppressing genes. Epigenetic silencing represents a promising approach to induce cytotoxicity in ATC. Broadly, two categories of epigenetic modulators have been tested: Histone Deacetylases (HDACs) inhibitors and Bromodomain and Extra-Terminal (BET) inhibitors. Trichostatin-A (TSA) and Suberanilohydroxamic (SAHA or Vorinostat) are two well-characterized HDAC inhibitors, which restored the expression of the thyroid-specific genes including *NIS, TSHR, TPO, TG,* and *TTF-1* in ATC cells and increased their radioiodine uptake [68]. Treatment with these HDAC inhibitors also showed diminished CD33 expression and increased expression of *NIS*, *Tg,* and *TTF1* in ARO cells. However, these HDACs inhibitors resulted in increased expression of stem cells markers *Oct4*, *Nanog*, *Sox2*, *Klf4,* and *c-Myc,* suggesting significant off-target effects [69]. Several other HDAC inhibitors exhibited promising results without such side-effects. Thailandepsin A (TDP-A) showed promising antiproliferative effects concomitant with cell cycle arrest and apoptosis activation in ATC cells [70]. N-hydroxy-7-(2-naphthylthio) heptoxide (HNHA) is a recently discovered HDAC inhibitor that showed promising results in PTC and ATC cell lines by inducing caspase-dependent and ER stress-mediated apoptosis [71]. Similarly, Pugliese et al. showed that treatment with LBH589 can induce re-differentiation in the ATC cell lines BHT-101 and Cal-62, marked by an increased NIS expression and radioiodine uptake [72]. This class of inhibitors warrants further investigation to explore the therapeutic implications in preclinical and clinical settings.

A recently emerging category of epigenetic modulators, BET inhibitors exert their biological function by targeting the bromodomain and extra-terminal of BET proteins, which interact with HDACs and regulate gene expression. Two recently discovered BET inhibitors, JQ1 and I-BET762 blocked cell cycle arrest in ATC cells by targeting MCM5. MCM5 is highly over-expressed in PTC and ATC tissue specimens indicating its potential as a molecular target [73]. In particular, JQ1 was evaluated in an ATC mouse model, ThrbPV/PVKrasG12D, and exhibited significant tumor reduction and improved survival which is mediated by reduced *MYC* expression and disrupted cyclin-CDK4/RB/E2F3 signaling, indicating its promising applications as an anti-cancer drug [74].

### Metabolic pathway targeting

Cancer cells have a high proliferation rate and altered metabolomic landscape which can be employed in therapeutics. Very few studies have been done towards targeting metabolomic pathways in ATC. A glucose analog, 2-deoxyglucose (2DG) was shown to re-sensitize ATC cells to radiation and chemotherapy (Cisplatin), but the observed effect was transient [75]. Also, an analog of vitamin D3, 19-nor-2α-(3-hydroxypropyl)-1α,25-dihydroxy vitamin D_3_ (MART-10) was demonstrated to inhibit migration and invasion of ATC cells by blocking the EMT pathway [76]. Microarray analysis of ATC versus normal thyroid tissues revealed significant distortion of fatty acid metabolism, and Stearoyl-CoA desaturase 1 (SCD1) was identified as a differentially expressed enzyme in ATC. SCD1 targeting induced endoplasmic reticulum stress and consequently apoptosis in ATC cells, both in vitro and in vivo [77]. Hence, this approach might help in improving the existing therapeutic interventions utilized today.

### Apoptosis enhancing strategies

Cancer cells bypass apoptotic signals and often display insensitivity to apoptosis-inducing agents. In thyroid cancer, an important apoptosis-inducing molecule, TNF-related apoptosis-inducing ligand (TRAIL), has been shown to potently and selectively kill cancer cells. TRAIL has emerged as an attractive molecular target owing to its cancer cell specificity and lack of toxicity to normal cells. TRAIL resistance factors include activation of c-FLICE-like inhibitory protein (c-Flip) and reduced expression of Trail-R1 and Trail-R2 receptors on the tumor cell surface. Interestingly, gene silencing of c-Flip and MADD, a key player in TRAIL-induced apoptosis, can significantly improve the TRAIL sensitivity [78]. Further, *MADD* knock-down and/or MADD dephosphorylation can also render differentiated thyroid cancer cells susceptible to TRAIL [79]. Thus, targeting these TRAIL resistance factors can be used to improve TRAIL sensitivity. In an independent study conducted by Gunda et al., the TRAIL-R2 receptor agonistic antibody, Lexatumumab, was shown to induce apoptosis in HTH7 (ATC), BCPAP and TCP-1 cells. Interestingly, in Lexatumumab resistant cells harboring a BRAFV600E mutation, a combination of BRAF inhibitor (PLX4720) or PI3k inhibitor (LY294002) can be employed to overcome apoptotic resistance [80]. Several HDAC inhibitors were shown to synergize with TRAIL activity and can be employed to overcome its resistance. For instance, combining TRAIL with the HDAC inhibitor Vorinostat (SAHA) resulted in increased DR5 expression and cell death [81]. Combination of TRAIL and the HDAC inhibitor VPA also induced significant apoptosis in TRAIL-resistant 8505C (ATC) cells by activating Jnk and phosphorylating FADD and c-jun, but not p38 [82]. HDAC inhibitors such as SAHA and MS-275 promoted apoptosis by preventing TRAIL degradation in thyroid cancer cells [83]. Another apoptosis-inducing agent, Obatoclax (BCL inhibitor) induced significant cell death through necrosis and lysosome neutralization in ATC cells [84].

### Immunotherapy

The presence of TAMs, NK cells and other TILs within ATC tissues highlight the relevance of tumor-immune cell interaction [85]. TAMs (type M2) promote tumor growth in ATC by expressing high levels of immunosuppressive cytokines such as IL-10 and TGF-β1 [86]. Other immunosuppressive mechanisms include binding of Programmed Death Ligand-1 (PD-L1) with its cognate receptor PD1 expressed on T cells, which down-modulates effector T cell function. In ATC, BRAFV600E mutation is strongly associated with the expression of PD-L1 (*P* = 0.015) [87]. In a recent retrospective study, high PD-1 expression (> 40% staining) in inflammatory cells was associated with worse overall survival (OS; hazard ratio, 3.36; 95% confidence interval, 1.00 to 12.96; *P* < 0.05) and trended towards worse PFS, whereas high PD-L1 expression in tumor cells (> 33% staining) trended towards worse PFS and OS indicating the crucial role of PD-1/PD-L-1 pathway in ATC [88]. In a preclinical BRAF^V600E/WT^;p53^−/−^ mouse model, treatment with a BRAF inhibitor (PLX4720) and an anti-PD-L1 antibody resulted in a significant tumor regression and strong anti-tumor immune response [89]. The potential use of immunotherapy was exemplified by an exceptional response observed upon treatment with Vemurafenib (BRAFV600E inhibitor) and Nivolumab (human IgG4 anti-monoclonal PD-1 antibody) for tumor harboring BRAF mutation and PDL-1 positivity [90]. Several clinical trials using inhibitors/antibodies targeting PD-1 and PD-L1 are underway for clinical trials in ATC patients (Table 2) and hold promise.

Like PD-1/PD-L1, another crucial tumor-immune cell interaction that can be a potential immunotarget for ATC is CD70-CD27 as it is found in 49% of ATC specimens [91]. A clinical study showed that CD70 expression was associated with BRAFV600E mutation in ATC lesions and remained stable throughout the disease progression. However, no correlation was observed between CD70 and PD-L1 in ATC [91]. In addition, NK cell-based adoptive cellular therapy showed promising results in a preclinical mouse model of ATC pulmonary metastasis [92]. The major hurdle with immunotherapy is the low number of TILs, thus agents that can improve TIL trafficking needs to be explored.

### Combination treatment

Owing to inherent and acquired chemoresistance, a combination of different drugs is often used in preclinical and clinical trials to improve therapeutic efficacy. However, it is important to establish a synergistic relationship between two drugs for their implications as a combination therapy**.** Several investigations were conducted to determine the relationship between drugs and some of them revealed crucial information about their behavior in combination. In this context, Allegri et al. have shown a synergistic effect between a CDK inhibitor (BP-14) and a mTOR inhibitor (Everolimus) by demonstrating loss of cell viability and down-regulation of EMT-related genes [93]. Combination of the NF-κB inhibitor (Quinacrine) and Sorafenib showed improved survival in an orthotopic mouse model in comparison to vehicle-treated and Doxorubicin-treated mice [94]. Combining MEK inhibitor (Trametinib) and multi-kinase inhibitor (Pazopanib) showed a significant reduction in the growth of xenografted tumors containing KRASG12R and BRAFV600E mutations [95]. Treatment with a combination of Carboplatin (CBDCA) and Radachlorin-photodynamic therapy (PDT) resulted in a significant tumor reduction due to activation of intrinsic apoptosis [96]. Combination of BRAF inhibitor (PLX4720) and Src tyrosine receptor/Bcr-Abl family inhibitor (Dasatinib) showed reduced tumor size, increased immune cell infiltration and induced apoptosis in an orthotopic ATC mouse model [97]. Combining PPARγ ligand (Troglitazone) and cholesterol-lowering drug (Lovastatin) demonstrated a significant suppression of EGF-induced migration in ATC cells, marked by the reduction of Vimentin and N-cadherin [98]. Synergistic cytotoxicity with Doxorubicin and Cucurbitacin B was observed in ATC cells in vitro and this effect *was* modulated by JNK2/STAT3 and ERK1/2 [99]. However, this effect is yet to be demonstrated in vivo. A combination of HDAC inhibitor (SAHA) and the PARP inhibitor (PJ34) exhibited a synergistic effect against SW1736 cell growth in vitro. This combination treatment also caused induction of *TSHR,* but not of *NIS*, *TTF1*, *TTF2*, and *PAX8* mRNA levels [100]. Similar synergistic effects were observed with HDAC inhibitor (PXD101) and HSP90 inhibitor (NVP-AUY922), concomitant with the inactivation of PI3k/Akt signaling and activation of DNA damage response in ATC cells [101]. An HSP90 inhibitor, SNX5422 revealed synergy with many HDAC inhibitors including PXD101, SAHA, and TSA [102]. Combination of CUDC-101 with a second-generation proteasome inhibitor, Carfilzomib, yielded synergistic effect by affecting cell cycle at G_2_/M phase and activating apoptosis depicted by PARP cleavage and Caspase-3 activation [103].

In contrast, some of the drug combinations exhibited an antagonistic or non-synergistic relationship. For instance, combining NF-κB inhibitors with taxane cytotoxic drugs and/or radiation therapy did not show any synergistic effect in ATC cells [104]. Similarly, a Pan MEK inhibitor (U0126) and BRAF inhibitor (PLX4720) did not show any inhibition of invasive potential of ATC cells suggesting that migration and invasion in ATC cells are mediated by other non-MEK mechanisms [104]. Hence, careful selection of a combination of drugs based on genomic and proteomic profiling of tumors is crucial for strategically improving the therapeutic efficacy.

## Conclusion

**Fig. 2:**
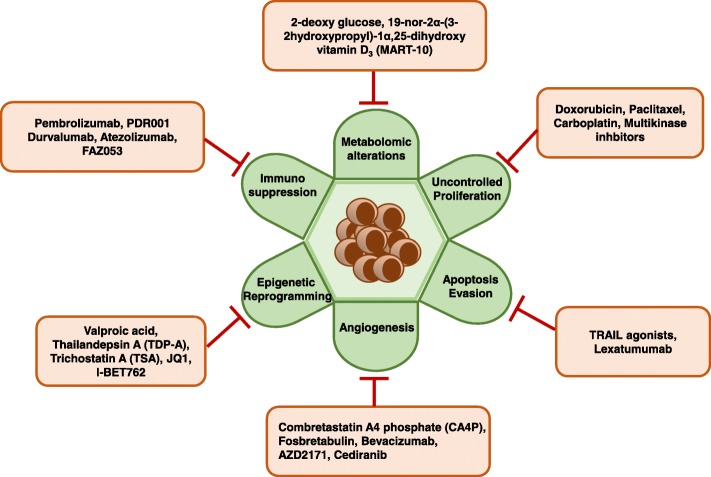
Different promising drugs/inhibitors that target several hallmarks of ATC including uncontrolled proliferation, resistance to apoptosis, immunosuppression, Epigenetic reprogramming, angiogenesis and metabolomic alterations

ATC remains a clinical challenge because of its de-differentiated phenotype and highly aggressive features. Several pre-clinical therapeutic studies including combinations of MKIs and HDAC inhibitors have shown encouraging results and hold promise for further investigation in clinical trials. Several clinical studies are ongoing to determine the safety and efficiency of novel drugs, but low patient accrual and limited long-term survival limit their translational ability. Combining several MKIs or using different salvage therapies might improve therapeutic outcomes. Evaluating drug treatment responses in primary cell cultures of patient tumors might help in guiding second-line treatment to develop precision medicine. An excellent case of such personalized therapy was demonstrated by Eckhard et al., wherein a patient’s tumor cells were cultured with Sorafenib, Vandetanib and MLN8054 (Aurora kinase inhibitor) in vitro while the patient was undergoing radiation and chemotherapy (Docetaxel and Cisplatin). Based on the in vitro data, the patient was subsequently treated with Sorafenib and achieved 43-month disease-free survival [105]. Analyzing patient biopsies during treatment might guide in understanding the mechanism of ATC progression, drug sensitivity, and chemoresistance leading to the selection of appropriate second-line of treatment. This can be exemplified by an unusual ATC case report in which an extraordinary response was achieved with Everolimus (FDA approved mTOR inhibitor) for the first 18 months with subsequent development of progressive disease. Comparison of the genomic sequences of sensitive tumors and drug-resistant tumors from the same patient revealed a nonsense mutation in *TSC2* (a negative regulator of mTOR), which imparted drug sensitivity. The acquired resistance to Everolimus was due to a mutation in the *mTOR* gene which prevented binding of Everolimus to mTOR (allosteric inhibition). However, mutated mTOR could still be targeted by other direct inhibitors [106]. This substantiates the fact that comprehensive genomic analysis of serial biopsies during the treatment might help in deciding the follow-up treatment strategy in an effective manner (Fig. 2).

Additionally, gaining insights into the tumor-specific mutational landscape will not only help in developing a tailored treatment plan but also paves the way to design novel therapeutics. Although the presence of TAMs within ATC substantially hinders the capturing of ATC specific transcriptomes via Next-Generation Sequencing, significant information can be derived by using this method. Also, there is a need to develop better deep sequencing and bioinformatic algorithms that account for TAMs background noise. Undoubtedly, assessment of cellular, genomic, and molecular data is critical to developing better diagnostic and therapeutic approaches to this disease. Development of precision medicine will benefit from comprehensive analysis of pharmacological markers to predict the course of treatment. Including such efforts might help better manage this lethal malignancy.

## Abbreviations

ALK: Anaplastic Lymphoma Kinase
ATA: American Thyroid Association
BET: Bromodomain and extra-terminal containing protein
CDK: Cyclin-dependent kinase
DTC: Differentiated Thyroid Cancer
EGFR: Epidermal Growth Factor Receptor
FDA: Food and Drug Administration
FGFR: Fibroblast Growth Factor Receptor
FLT-3: FMS Related Tyrosine Kinase 3
FNA: Fine Needle Aspiration
HDAC: Histone Deacetylase
Hsp90: Heat Shock Protein 90
HSPs: Heat Shock Proteins
LPEI: Linear Polyethylenimine
NIS: Sodium iodide Symporter
ORR: Objective Response Rate
OS: Overall Survival
PARP: Poly ADP ribose polymerase
PD-1: Programmed Cell Death Protein-1
PDGFR: Platelet-Derived Growth Factor Receptor
PDL-1: Programmed Death Ligand-1
PEG: Polyethylene Glycol
PLGA: Poly-lactic-co-glycolic acid
PLK-1: Polo-like kinase-1
PPARγ: Peroxisome Proliferator-Activated Receptor gamma;
PR: Partial Response
PTC: Papillary Thyroid Cancer
PTDC: Poorly Differentiated Thyroid Cancer
RR: Response Rate
SAHA: Suberoylanilide Hydroxamic Acid
TAM: Tumor-Associated Macrophages
TDP-A: Thailandepsin A
TG: Thyroglobulin
TPO: Thyroid Peroxidase
TRAIL: TNF-Related Apoptosis-Inducing Ligand (TRAIL)
TSHR: Thyroid-Stimulating Hormone Receptor
TTF-1: Thyroid Transcription Factor-1
TTP: Time to Progression
UICC: Union for International Cancer Control
VE-Cadherin: Vascular Endothelial Cadherin
VEGFR: Vascular Endothelial Growth Factor Receptor
VPA: Valproic acid
